# Case for diagnosis. An exophytic plaque on the chest. Carcinoma of the mammary crease^☆^^☆☆^

**DOI:** 10.1016/j.abd.2021.04.013

**Published:** 2021-09-30

**Authors:** Sonsoles Yáñez-Díaz, Marcos A. González-López

**Affiliations:** Dermatology Service, Hospital Universitario Marqués de Valdecilla, University of Cantabria, Cantabria, Spain

**Keywords:** Breast neoplasm, Carcinoma, skin appendage, Neoplasm metastasis

## Abstract

Carcinoma of the mammary crease is a very rare variant of breast carcinoma, in which the skin lesions are usually the presenting sign. The authors present the case of an 88-year-old woman with an exophytic plaque in the mammary crease of approximately ten years duration. The histopathological and immunohistochemical studies confirmed the diagnosis of infiltrative breast carcinoma (carcinoma of the mammary crease variant). This case highlights the important role of the dermatologist in the early diagnosis of this rare variant of breast cancer.

## Case report

**Figure 1:**
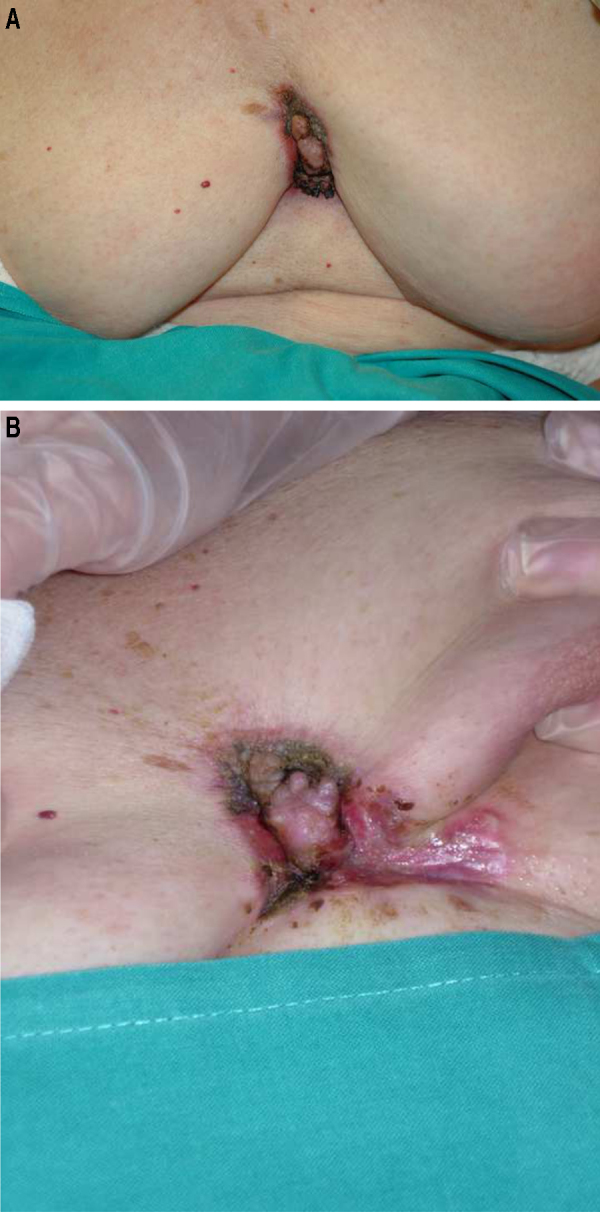
(A and B), Clinical Image. A 6 × 4 cm, infiltrated, erythematous-purple plaque in the median mammary crease, spreading to both inframammary creases and upper abdomen.

An 88-year-old woman was referred to our Department for evaluation of a cutaneous lesion on the chest. The lesion first appeared approximately 10 years ago and had gradually grown over time. She reported that the lesion was asymptomatic, although it bled several times in the last months. Dermatological examination revealed an exophytic, firm, infiltrated, erythematous-purple plaque measuring 6 × 4 cm in the median mammary crease, spreading to both inframammary creases and upper abdomen (Fig. 1). The lesion had well-demarcated borders with a scar-like appearance. There were no adenopathies and breast nodules were not palpable.

**Figure 2:**
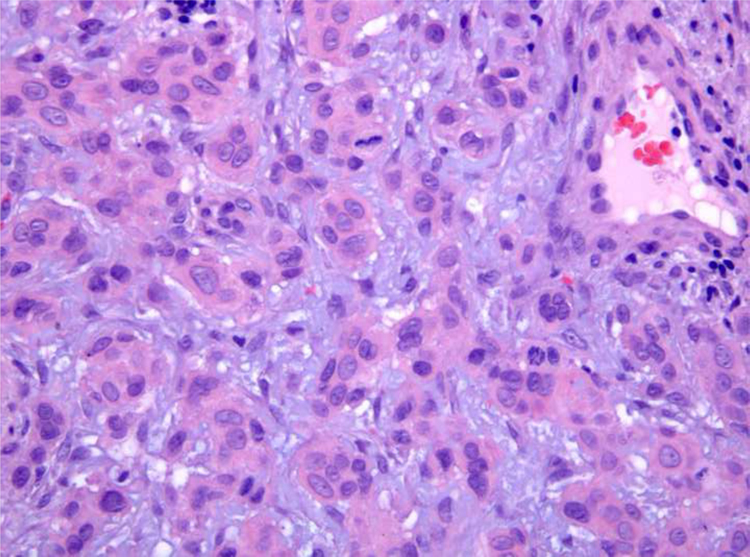
Histopathological examination. Neoplastic cells within a myxoid stroma (Hematoxylin & eosin, ×200).

**Figure 3:**
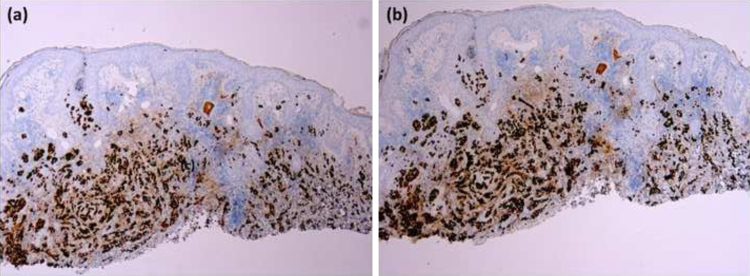
Immunohistochemical staining of the lesion. (a), Positive immunostaining for estrogen receptors (×20). (b), Positive immunostaining for progesterone receptors (×20).

A biopsy specimen revealed a dermal infiltrate of atypical cells which formed small solid nests within a myxoid stroma. The tumor cells exhibited large nuclei, prominent nucleoli and abundant eosinophilic cytoplasm (Fig.2). Immunohistochemically, the neoplastic cells were positive for epithelial membrane antigen (EMA), estrogen receptors (ER) and progesterone receptors (PR) (Fig. 3), and Bcl-2, but were negative for c-erbB-2.

## What is your diagnosis?

a) Basal cell carcinoma
b) Dermatofibrosarcoma protuberans
c) Carcinoma of the mammary crease
d) Squamous cell carcinoma

The patient was treated with radiotherapy and hormone therapy with tamoxifen with satisfactory results. However, four years later, she was admitted with respiratory failure and progressive deterioration of the general state. A chest X-ray and CT scan showed pleural effusion and bilateral nodular pattern compatible with metastatic spread, and the patient died two months later.

## Discussion

Carcinoma of the mammary crease (CMC) is an unusual variant of breast carcinoma, in which the skin lesions are usually the presenting sign.^1^, ^2^ The true incidence of CMC is difficult to estimate, although it may represent about 1% of breast cancers. Despite skin involvement being a striking feature of CMC, it has been very rarely described in dermatology journals.

The early skin involvement in this particular variant of breast cancer would be related to the anatomical characteristics of the inframammary fold that would explain the tendency of the tumor to invade the dermis or the underlying muscle.^3^, ^4^ Moreover, in CMC, the breast nodule is not palpable, and it is difficult to detect in mammography because of its peripheral location; therefore, skin manifestations are usually the initial reason for consultation in these patients.

Clinically, CMC may present itself as an ulcerated nodule or as a plaque, polypoid or verrucous lesion, and can simulate an inflammatory lesion, a benign tumor, or a cutaneous carcinoma.^1^, ^2^, ^4^, ^5^, ^6^ To this respect, confusion with morpheaform or ulcerated basal cell carcinoma is frequent due to the clinical appearance and chronic course of the lesion, even after histopathological examination.^2^, ^5^, ^6^ In these cases, only complete extirpation and/or immunohistochemical study, as in the case presented herein, allow a definitive diagnosis.^2^, ^6^ In the present study’s case, immunostaining showed positivity for ER, PR, and Bcl-2, but negativity for c-erbB-2, corresponding to a luminal A subtype of breast cancer.

In summary, this case highlights the prominent role of the dermatologist in the early diagnosis of CMC, which can contribute significantly to an increase in the survival of these patients.

## Financial support

None declared.

## Authors’ contributions

Sonsoles Yáñez-Díaz: Approval of the final version of the manuscript; elaboration and writing of the manuscript; intellectual participation in propaedeutic and/or therapeutic conduct of studied cases; critical review of the literature; critical review of the manuscript.

Marcos A. González-López: Approval of the final version of the manuscript; elaboration and writing of the manuscript; critical review of the manuscript.

## Conflicts of interest

None declared.
